# Communicative Rationality and Clinical Decision‐Making: A Habermasian Perspective on the Doctor–Patient Relationship

**DOI:** 10.1111/jep.70555

**Published:** 2026-09-13

**Authors:** Antonio da Silva Menezes

**Affiliations:** ^1^ Medicine Faculty Federal University of Goiás Goiânia Brazil; ^2^ Medical Sciences and Life Pontifical Catholic University of Goiás Goiânia Brazil

**Keywords:** communicative action, doctor–patient relationship, electronic health records, medical education, medical humanities, narrative medicine, patient‐centred care, shared decision‐making

## Abstract

Clinical encounters are where biomedical knowledge, institutional norms, and patients' lived experiences meet. Yet, modern healthcare structures these encounters through documentation, performance measures, and digital tools. These systems prioritise efficiency and measurable outcomes, often creating barriers to authentic dialogue between clinicians and patients. This tension raises philosophical questions about what conditions are necessary for meaningful clinical interactions. This article analyses the doctor–patient relationship using Jürgen Habermas' theory of communicative action. It argues that the key dynamic in clinical consultation is the negotiation of validity claims: truth (biomedical facts), normative rightness (institutional obligations), and sincerity (subjective experience). On this basis, the article proposes a conceptual model that characterises consultation as the interaction among three domains – biomedical facts, institutional norms, and patients' lived experience – summarised as ‘Facts, Norms, and Lives.’ The analysis studies how modern healthcare pressures shift clinical communication from dialogue aimed at mutual understanding to interaction driven by instrumental goals. The article argues that communicative methods – such as narrative competence and collaborative decision‐making – not only restore deliberative legitimacy in clinical encounters, but additionally reinforce the normative foundations of medical practice by conceptualising the consultation as a communicative structure.

## 1 Introduction

A clinical consultation is a small social institution. In a cramped exam room, the physician's eyes flick momentarily from the glowing computer screen to the patient's face, the low hum of ceiling lights combining with hurried keystrokes. Two people, often strangers and unequally positioned in power, knowledge, and vulnerability, try to agree on what is happening in the body, what it means in a life, and what should be done next. The encounter is epistemic (we need to know what is going on), ethical (we need to decide what is right), and political (we need to negotiate roles, authority, and legitimacy). When it works, it appears deceptively simple: the physician listens, the patient speaks, uncertainty is named, and a plan emerges. When it fails, the problem is rarely only ‘poor communication.’ Usually, it is a failure of mutual understanding, with consequences such as nonadherence, distrust, moral distress, and, at times, litigation [1].

**Figure 1:**
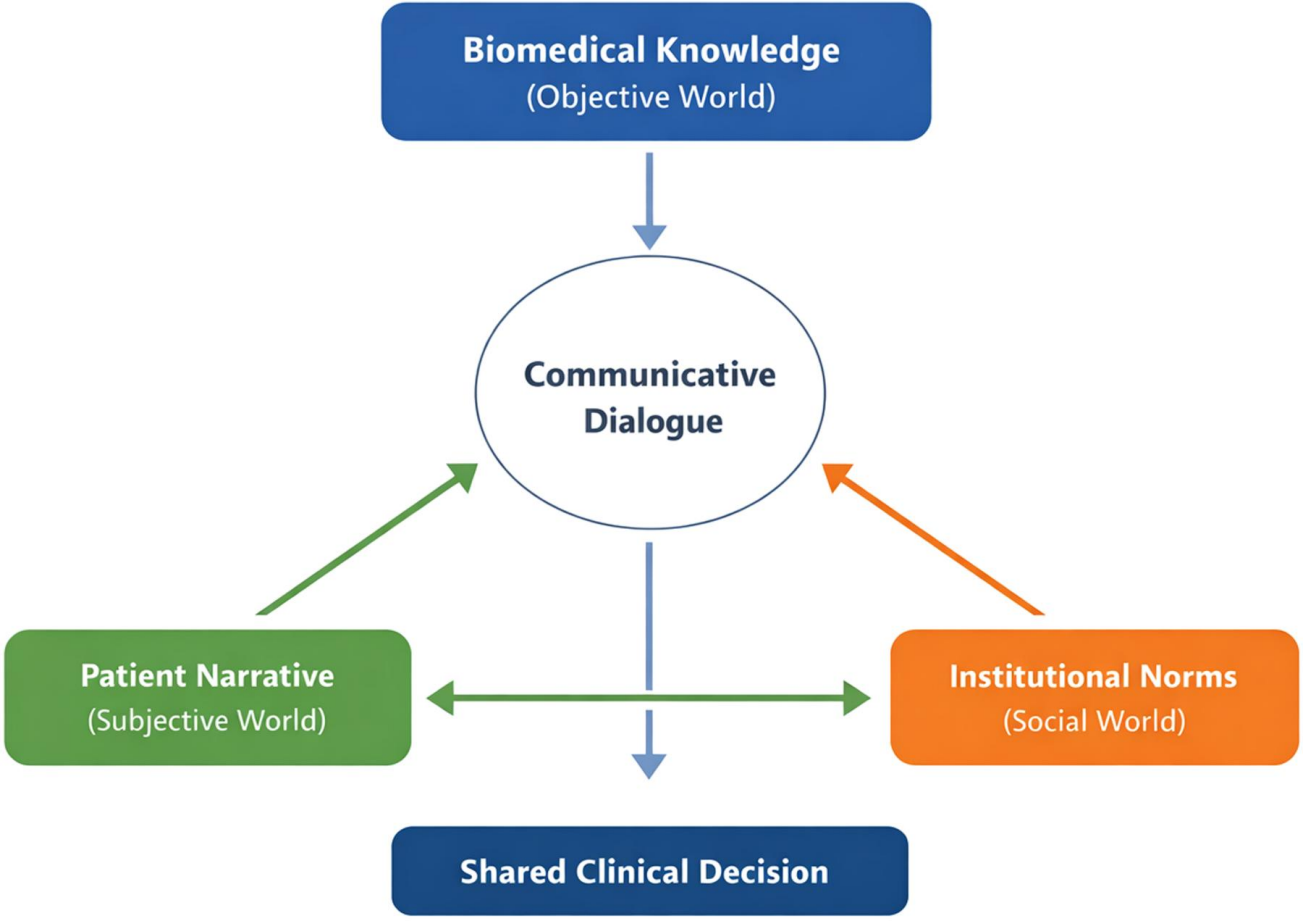
Communicative structure of the clinical encounter.

Healthcare research has accumulated evidence that clinician–patient communication matters for outcomes [1, 2, 3]. The critical question is: what is communication for? Many curricula treat communication as a technique: ask open‐ended questions, use empathy statements, and summarise. Technique is necessary; it is also insufficient. Consider a student who, during an assessment, recites, ‘I understand this must be difficult for you,’ before immediately moving to the next checklist item. This is different from a moment when a clinician pauses, responds to the patient's statement of fear, and, together, they reconsider the plan, weighing uncertainties and seeking shared ground. The first enacts empathy as a scripted performance; the second enacts competence in making meaning together. Without a theory of communicative practice, ‘communication skills’ risk becoming a checklist performance in which students learn to sound empathic while the structural conditions of the encounter make genuine deliberation improbable [4, 5, 6], as shown in Figure 1.

The consultation has become crowded. Patients bring narrative symptoms, disruptions, fears, interpretations, and social constraints [7, 8]. Clinicians bring diagnostic frames, evidence, and guideline pathways. Increasingly, the documentation system joins as a third participant. Time‐and‐motion studies show that, in ambulatory practice, physicians spend substantial time on Electronic Health Records. (EHRs) and desk work compared with time spent with patients [9]. The EHR does not simply serve as a tool; it acts as a social actor, shaping gaze, turn‐taking, and what counts as ‘relevant’ information. Of course, this agency is not inherent to the software itself but is programmed, mandated, and reinforced by human actors, including administrators, policymakers, and institutional leaders who set priorities and design workflows. Noticing these decision‐makers prevents technology from seeming autonomous and reminds us that the social effects of the EHR are continuously negotiated [10, 11, 12, 13, 14, 15].

The medical humanities have long argued that modern institutions distort human relationships by replacing dialogue with procedure. Jürgen Habermas offers a vocabulary for this distortion. He distinguishes instrumental rationality – action‐oriented toward success, control, and optimisation from communicative rationality, interaction‐oriented toward mutual understanding [16]. Instrumental rationality is concerned with achieving outcomes efficiently, while communicative rationality seeks to reach consensus and understanding through dialogue. Clinical medicine undeniably needs instrumental rationality: tests, protocols, risk prediction, and procedural skill. The danger is not instrumentality itself, but its expansion into spaces that should be governed by communicative action. Habermas calls this the ‘colonisation of the lifeworld’ by system imperatives [16, 17]. In healthcare, these imperatives appear as throughput, coding, documentation, productivity metrics, and standardised pathways. To illustrate the contrast, consider physician reimbursement tied to completing specific electronic health record documentation and meeting performance standards, such as the percentage of patients receiving certain screenings. In such cases, organisational priorities grounded in instrumental rationality may override opportunities for communicative interaction [16]. As a result, clinicians may feel compelled to structure their patient encounters to meet these system priorities, sometimes at the expense of open‐ended dialogue and patient concerns. This article asks: what happens to the doctor–patient relationship when these imperatives reshape the encounter's internal logic? [16, 17].

We have four claims. First, the clinical encounter is a communicative situation in which participants raise and negotiate validity claims – truth, rightness, sincerity under conditions of asymmetry and time pressure [16]. Second, contemporary systems risk turning encounters from communicative to strategic: patients are managed rather than understood; consent is obtained rather than deliberated. Third, narrative medicine and medical humanities offer pedagogical approaches to restore communicative rationality by training clinicians to interpret stories, attend to metaphors and silences, and reflect on power [6, 7, 8, 17]. Fourth, Brazil's national educational policy offers an explicit mandate: the 2025 DCNs for Medicine define the physician as an important, reflective, humanistic professional, emphasising the importance of qualified listening and person‐centred care [18, 19]. The policy question is curricular: how can medical schools enact these commitments, so that ‘humanism’ serves as an organising logic rather than an isolated module?

## 2 The Crisis of Dialogue: Why “More Empathy” Is Not Enough

When patients say ‘the doctor didn't listen,’ they often mean something specific: their narrative failed to reorganise the clinician's agenda. One patient recalled, ‘I seemed like a case to be managed, not a person to be known. My words did not change the outcome.’ The clinician may have allowed them to speak, but their account did not become consequential. Conversely, rushed clinicians often report that they did listen but had to ‘get through’ required items: a template, safety cheques, a quality metric, and a discharge summary. Both perspectives can be true. The crisis goes beyond interpersonal issues; it is structural.

Consider a typical vignette. A 58‐year‐old woman with diabetes presents for a follow‐up. The EHR flags overdue vaccinations, eye exams, A1c cheques, and depression screening. The clinician has 12 min. The patient opens with, ‘Doctor, I'm exhausted.’ The clinician hears fatigue; the template demands screening items. The clinician asks depression questions; the patient answers minimally. This reserved, clipped speech is not simply an absence; the patient's silence is itself a narrative clue, a form of data pointing to unspoken concerns or discomfort. A narrative‐competent listener would pause to consider what the patient's withholding might signify: overwhelm, mistrust, or perhaps distress that cannot be easily voiced. Instead, the clinician orders labs, adjusts medications, and prints education materials. The patient leaves with a plan but without feeling understood. Nothing ‘wrong’ occurred. Yet the encounter failed as a communicative practice: the patient's lifeworld work stress, caregiving burden, and fear did not enter the reasoning. The care is clinically competent but existentially thin.

Research suggests that certain communicative practices, such as turn‐taking, pausing, agenda‐setting, and soliciting questions, support ‘unhurried conversations’ even under constraints [20]. But even these points are deeper: unhurriedness is not just time; it's the moral readiness to be interrupted by the patient's meaning. If the system punishes interruption‐rewarding throughput, penalising complexity ‘be more empathic’ becomes a moral burden on clinicians rather than a systems issue [21].

The EHR illustrates the ambivalence of technology. Some studies note that EHR‐related pauses can be welcomed by patients as opportunities to participate [12]. Yet other analyses show that multitasking can reduce eye contact and divide attention, undermining patient‐centred communication [22]. These findings are not contradictory; they show that the EHR can be used in different ways depending on workflow, training, and administrative priorities. Rather than adopting a simple stance for or against digital documentation, it may be more productive to ask: under what conditions does an EHR pause support dialogue, and under what conditions might it become an obstacle? By surfacing these dual possibilities, we invite clinicians and educators to consider their own practice. The humanities' contribution is to illuminate what the EHR does to discourse: what becomes sayable, what becomes invisible, what becomes measurable.

A second vignette illustrates strategic distortion. A patient with atrial fibrillation (AF) is offered anticoagulation. The clinician quickly explains risks and benefits. The patient nods: consent is ‘obtained.’ The chart notes, ‘discussed and patient agrees.’ Yet the patient's fear of bleeding, lifestyle disruption, or being ‘on a drug forever’ remains unexplored. Shared decision‐making would require deliberation about values and trade‐offs [23, 24, 25]. The encounter becomes strategic: the clinician aims for therapy uptake; communication becomes persuasion disguised as education. The patient may later discontinue medication ‘non‐adherently,’ which is then framed as patient failure rather than communicative failure.

## 3 Habermas' Framework: Validity Claims, Lifeworld, and System

Habermas begins with an insight that is surprisingly practical for clinicians: everyday communication works because speakers implicitly raise claims that can be challenged and defended. In communicative action, participants orient toward mutual understanding; they assume that reasons can matter [16, 26]. Habermas identifies three core validity claims. To explain these for clinical readers, we can translate them into the language commonly spoken at the bedside, as follows [27]:

- Truth: claims about facts. In clinical settings, this can sound like, ‘Based on your symptoms and test results, this is what we know so far,’ or, from the patient, ‘My pain gets worse when I walk.’
- Rightness: claims about norms. Clinicians might use the language, ‘It's important that you feel you can speak openly here,’ or ‘You have a right to understand your options and decide what's best for you,’ while patients might say, ‘I want to be part of this decision,’ or ‘I expect honesty and respect.’
- Sincerity: claims about subjective experience. At the bedside, a patient may share, ‘I am really worried about what this means for my life,’ and the clinician might respond, ‘I truly want to help you find a plan that fits your needs.’

By connecting these validity claims to familiar clinical phrases, we see how these abstract philosophical concepts are enacted in every consultation. Clinical encounters constantly negotiate these claims. Biomedical reasoning evaluates truth claims through evidence, tests, and probabilities. Ethical practice evaluates rightness claims considering consent, fairness, and professional duties. Therapeutic relationships depend on sincerity claims: whether the patient feels safe enough to disclose, whether the clinician's empathy is perceived as genuine.

Habermas also distinguishes lifeworld and system. Lifeworld is the background of meanings that make communication possible: shared culture, biography, and tacit norms. Illness is experienced in the lifeworld: it disrupts roles, identity, time and family. The system is the institutional coordination mechanism: hospitals, billing, legal rules, and protocols [16]. Modern societies need systems. The problem arises when system logic displaces lifeworld communication. Habermas calls this colonisation: the replacement of understanding‐oriented discourse by mechanisms of money and power [28].

In healthcare, colonisation can appear in several practical forms. Template dominance emerges when clinical documentation systems prioritise what can be recorded rather than what is meaningful to patients. Metrics dominance occurs when quality indicators and performance measures redefine care in terms of measurable outputs. Compliance framing shifts attention toward whether patients follow prescribed plans rather than whether those plans align with their lived circumstances. Finally, risk‐language dominance reduces the patient to a set of probabilistic risk estimates rather than to a person experiencing illness. These dynamics reflect how institutional systems and managerial logics may reshape the communicative space of the clinical encounter, privileging instrumental rationality over deliberative dialogue [14, 16].

Crucially, colonisation is not only imposed; it is internalised. Clinicians begin to talk and think in system language. They may describe a patient as ‘noncompliant,’ ‘frequent flyer’ or ‘social admission.’ These phrases are not simply rude; they are system categories that compress complexity into workable labels. They perform moral work by justifying the limitation of dialogue. When was the last time you used or accepted one of these terms in your own clinical or teaching practice? A moment of self‐audit can turn abstract critique into personal insight, making visible how these reductions enter our own speech and thought. A Habermasian critique, therefore, targets not individual bad actors but the discursive environment that makes certain reductions feel normal. The contrast summarised in Table 1 illustrates how these two rationalities shape different understandings of the physician–patient encounter. While instrumental rationality emphasises diagnostic efficiency and protocol‐driven care, communicative rationality foregrounds dialogue, interpretation and shared decision‐making.

**Table 1:** Instrumental and communicative rationality in clinical encounters.

Dimension	Instrumental rationality in clinical practice	Communicative rationality in clinical practice	Implications for medical education
The primary goal of interaction	Efficient diagnosis and management of disease	Mutual understanding of illness and collaborative decision‐making	Train students to balance diagnostic efficiency with deliberative dialogue
Orientation of action	Success‐oriented: achieving predefined clinical outcomes	Understanding‐oriented: reaching a shared interpretation of the situation	Teach reflective listening and narrative interpretation
Role of the physician	Technical expert applying biomedical knowledge	Dialogical participant integrating biomedical and experiential knowledge	Develop communicative competence, not only technical expertise
Role of the patient	Passive recipient of medical advice	Active contributor of experiential knowledge and personal values	Teach methods for eliciting patient narratives and values
Structure of communication	Question–answer format aimed at extracting biomedical information	Open dialogue integrating symptoms, meaning, fears, and preferences	Teach agenda‐setting and shared decision‐making
Dominant knowledge type	Biomedical evidence and clinical guidelines	A combination of biomedical knowledge and the lived experience of illness	Integrate evidence‐based medicine with narrative medicine
Use of technology	Digital systems structure the encounter and guide documentation	Technology is used as a shared informational resource supporting dialogue	Teach strategies for patient‐centred EHR use
Ethical orientation	Professional authority guiding treatment decisions	Shared deliberation respecting patient autonomy and values	Embed ethics within communication training
Risk in practice	Reduction of the patient to a clinical case or data set	Potential inefficiency or ambiguity in decision‐making	Teach negotiation of time constraints without abandoning dialogue
Educational focus	Procedural and technical competence	Communicative, interpretive, and reflective competence	Integrate medical humanities longitudinally in curricula

## 4 The Consultation as a Communicative Situation: Objective, Social, Subjective Worlds

From this perspective, the clinical encounter is a negotiation across three worlds: ‘Facts, Norms, Lives.’ [1] Objective world (biomedical facts): lab results, imaging, epidemiology, clinical probabilities [2]. Social world (norms and institutions): professional obligations, patient rights, organisational rules, cultural expectations of good care [3]. Subjective world (lived experience): fear, hope, suffering, values, biography. Coining the phrase ‘Facts, Norms, Lives’ helps trainees anchor the triad in memory and recall the need to consider each dimension in every encounter, as seen in Table 2.

**Table 2:** Communicative dimensions of the clinical encounter: integrating facts, norms and lives.

Dimension of the encounter	Description in clinical practice	Typical distortion under instrumental rationality	Communicative practice
Objective world (Facts)	Biomedical information: symptoms, physical findings, laboratory tests, imaging, epidemiology	Reduction of illness to pathophysiology alone; patient narrative treated as irrelevant	Translate biomedical evidence into language meaningful to the patient
Social world (Norms)	Institutional rules, professional responsibilities, clinical guidelines, and ethical duties	Policy or protocol replaces deliberation (“This is the guideline”)	Explicit negotiation of norms, rights, and responsibilities
Subjective world (Lives)	Patient experiences, fears, expectations, values, biography, and social context	Patient concerns are ignored or reframed as “non‐compliance.”	Active exploration of patient narrative and meaning
Integration across worlds	Deliberative process combining facts, institutional constraints, and personal values	Strategic communication aimed at compliance or throughput	Shared reasoning and patient‐centred decision making
Outcome of communication	A clinical plan grounded in both biomedical evidence and the patient's life context	Treatment plan disconnected from patient values or capacities	A feasible and legitimate care plan was developed through dialogue

Table 2 summarises how these three communicative dimensions operate within clinical encounters and how distortions emerge when instrumental rationality dominates.

A consultation fails when one world silences the others. A clinician reviews a patient's blood results, makes barely any eye contact, and increases the medication dosage without asking about recent changes or concerns. The visit ended quickly, and both participants disengaged. Biomedicine dominates if symptoms are reduced to pathophysiology, stripping them of narrative meaning. Norms dominate if rules replace deliberation: ‘This is the policy.’ Subjectivity dominates if facts are denied or evidence dismissed. Good medicine integrates all worlds, translating facts into meaning and meaning into decisions.

Consider a composite clinical scenario illustrating this triad. A 60‐year‐old man with poorly controlled hypertension visits the clinic with his daughter. The objective world appears: his blood pressure is high, and labs show early kidney damage. The social world surfaces in policy: assess adherence, reinforce advice, and practice shared decision‐making. The subjective world appears as the patient hesitates to start another medication, fears side effects, and discusses work and financial struggles. ‘I can't afford another pill, and I'm already tired all the time,’ he says, looking at his daughter. Instead of escalating medication (objective dominance) or echoing policy (normative dominance), the clinician explores the patient's worries and seeks the daughter's input on managing medications. They review treatment options, discuss side effects and costs, and agree on a low‐cost medication, scheduled follow‐up, and dietary support. Facts, institutional norms, and lived experience merge in a shared, feasible decision.

For educators designing simulations, these composite cases anchor the mix of biomedical facts, norms, and patient experience, showing trainees how concrete deliberation weaves together the three worlds without letting one dictate the outcome. To translate this curricular intent into assessable simulation objectives, facilitators can identify specific learner behaviours during the debrief. For example, trainees might elicit a patient value statement that brings the subjective world forward, reference a policy constraint aloud to demonstrate awareness of institutional norms, and explain clinical findings in accessible language to address the objective world. By articulating and observing actions like these, instructors can more clearly assess and guide learners through balancing Facts, Norms, and Lives.

Shared decision‐making aims to institutionalise communicative rationality in the clinic. Stiggelbout et al. argue that it truly ‘puts patients at the centre’ by aligning decisions with patient values, especially when there are multiple viable options [29]. To clarify, such preference‐sensitive choices tend to exist when:

− There are small or uncertain differences in expected health outcomes between options.
− The possible risks or benefits affect the quality of life or day‐to‐day functioning.
− Treatment options differ in mode of administration, duration, side effects, or impact on lifestyle.
− Patient preferences or values might reasonably lead to different choices.

In contrast, some decisions are more clinically mandated, such as when only one safe or effective intervention is available. Elwyn et al. present a practical model choice talk, option talk, and decision talk that explicitly invites patients into deliberation [30]. In Habermasian terms, shared decision‐making models exchange facts (truth), make partnership norms explicit (rightness), and elicit patient preferences (sincerity) [31].

But shared decision‐making is easily distorted. Under time pressure, it becomes ‘information dumping’: options are listed quickly, and the clinician strongly recommends one. The patient ‘chooses’ what the clinician prefers. This is a strategic action masked as communication. The problem is not persuasion itself; clinicians should recommend, but hide persuasion under the guise of neutrality. A more ethical posture is what the humanities call ‘honest recommendation’: being transparent about one's professional judgement while openly sharing reasons and preferences. Explicitly naming this stance can invite clinicians to reflect on their communicative intent. Embedding one simple question – ‘Have I declared my stake?’ can help convert humanities theory into regular practice. The humanities insist: if recommending, be clear; if options exist, invite debate; if options are limited, state constraints.

This communicative structure is especially important in contemporary clinical practice, where the consultation often extends beyond a simple dyadic physician–patient relationship. In ageing societies and complex care settings, clinical decisions must frequently take into account the patient's lifestyle, activities of daily living, quality of life, family context, caregiver participation, previously expressed wishes, and dignity. In situations involving dementia, unconsciousness, frailty, or impaired decision‐making capacity, communicative rationality should not be understood as requiring only the patient's immediate verbal participation. Rather, it requires a deliberative effort to reconstruct, as faithfully as possible, the patient's values, preferences, life history, and understanding of dignity through dialogue among physicians, family members, surrogate decision‐makers, caregivers, and multidisciplinary teams. From a Habermasian perspective, such deliberation preserves the normative orientation of clinical decision‐making toward mutual understanding, justification, and respect for those affected by the decision.

## 5 Medical Humanities and Narrative Medicine: Not Decoration, but Epistemology

In my internal medicine rotation, I once saw a patient, Mrs. L, who described her chest pain as ‘like a rope constricting around my heart.’ Focused on her normal test results, I almost dismissed her remarks as anxiety. Only when another doctor asked her to go into detail about her story did we uncover a recent grief that had triggered takotsubo cardiomyopathy – a diagnosis I had missed because I did not attend fully to her metaphor. The cost of missing her story was nearly missing her disease.

Medical humanities are often seen as enrichment, an addition to a biomedical curriculum. This framing is part of the problem. Humanities are not simply about ‘being nicer’; they are about how knowledge works in medicine. Patients do not present ‘data’; they present stories. Clinical reasoning begins as narrative reasoning: what changed, what is different, what matters, what cannot be ignored. To clarify the core distinction: treating narrative as clinical evidence means valuing what patients say as essential clues that directly influence diagnosis and management, more than just as bedside courtesy. Narrative medicine argues that narrative competence is a cognitive skill: the ability to recognise plot, causal attributions, metaphors, omissions, and turning points [6, 10, 31]. Importantly, narrative competence is not ornamental; it is epistemic [4]. The ability to notice a patient's metaphors, such as ‘it feels like a pressure bearing down on my chest,’ is linked directly to clinical rigour. What diagnosis did you almost miss because a metaphor slipped by? The stakes are both diagnostic and relational. Overlooking the narrative risks missing what is clinically essential. Reframing narrative attentiveness as a source of diagnostic acuity aligns the medical humanities with core clinical priorities. It prevents treating them as an optional add‐on.

Charon's papers make a central claim: narrative medicine is not only about empathy, reflection, professionalism, and trust [6, 10]. It is primarily an epistemic model – attending to narrative is required to truly know what is happening with a patient. For example, a patient's metaphor – ‘it feels like an elephant sitting on my chest’ – is diagnostic, not merely expressive. Likewise, a patient's silence after a diagnosis may signal something clinically significant rather than just emotion.

A reductionist colleague might argue, however, that narrative introduces subjectivity and distracts from the objective measurements which underpin medical science. From this perspective, relying on narrative risks leading clinicians astray or perpetuating diagnostic error. Yet even the most quantitative data requires interpretation within a context that narrative helps to provide. For instance, the stories patients tell can clarify the onset and trajectory of symptoms when lab values are equivocal or atypical. Rather than undermining rigour, narrative reasoning complements and strengthens evidence‐based practice, ensuring that clinical judgements are grounded in both empirical data and the realities patients convey.

Arthur Kleinman's classic argument in The Illness Narratives remains that illness is not just disease. It is a suffering formed by meaning and culture [21]. Clinicians who focus only on disease may miss the experiences that drive care‐seeking. Emanuel and Emanuel's 'four models' paper also shows how different physician–patient relationships embed different moral assumptions about authority and value [32]. The point is simple: communication is not neutral. It enacts a model of medicine.

The humanities also insist on analysing power. Patients differ in their resources and voices, and institutional inequalities shape which narratives are truly heard. For example, research shows that clinicians are less likely to take pain reports from Black patients as seriously as those from white patients, and language barriers can further obstruct the communication of symptoms and suffering [33]. Socioeconomic status also influences a patient's ability to advocate for themselves, affecting whose stories are considered credible [34]. A ‘patient‐centred’ approach can become a slogan unless it confronts inequalities. Mead and Bower's framework identifies dimensions of patient‐centredness, such as sharing power and responsibility and seeing the ‘patient as person.’ Yet those dimensions are not automatically achieved by training; they require institutional support and self‐critique [35].

The hidden curriculum is the mechanism through which institutions undermine formal humanistic teaching. Hafferty argues that curriculum reform often fails. The informal norms of training—what is rewarded, mocked, or ignored‐ shape identity more than lectures do [36]. A student may learn to say the empathy line in OSCEs. At the same time, they learn that emotions are obstacles to efficiency. In that case, communication teaching becomes performance. In my training, I recollect a moment during a clinical assessment. I delivered a precisely rehearsed phrase: I understand this is difficult for you. Yet, I was already thinking about my next checklist item. I sounded empathetic, but I was reciting a script instead of genuinely connecting with the patient. Caught between wanting to appear competent and feeling the pressure to move quickly, I experienced a subtle discomfort that something meaningful was missing, even as I performed well by external standards. I remember noticing a distance between my words and my intention, a quiet conflict that left me uneasy but professionally unnoticed. This moment appeared empty, but it passed without comment. No one questioned it, and I turned my focus to the next task. Only later did I realise how easily I internalised the expectation that efficiency and the appearance of sympathy mattered more than authentic engagement. Naming my complicity in this hidden curriculum may help others recognise comparable patterns in their own practice. Humanities pedagogy must be longitudinal and embedded in clinical settings, not isolated to pre‐clinical years, as demonstrated in Table 3.

**Table 3:** Educational strategies for developing communicative competence in medical training.

Educational domain	Pedagogical strategy	Learning objective	Observable assessment outcomes
Agenda negotiation	Simulated consultations with multiple competing concerns	Train students to negotiate priorities with patients under time constraints	Agenda‐setting statements, shared prioritisation of concerns
Narrative listening	Close reading of illness narratives and reflective discussion	Develop the ability to recognise metaphors, omissions, and turning points in patient stories	Identification of narrative elements in patient accounts
Shared decision‐making	Workshops using decision aids and structured dialogue models	Train students to elicit patient values and deliberate over treatment options	Demonstration of choice talk, option talk, and decision talk
Reflective practice	Parallel chart writing (clinical note + narrative reflection)	Encourage integration of biomedical reasoning with patient experience	Reflective writing demonstrating ethical and interpretive awareness
Critical awareness of system constraints	Case discussions analysing institutional pressures	Develop the capacity to recognise how system priorities shape communication	Student identification of workflow pressures and ethical tensions
Faculty development	Training clinicians to model communicative reasoning during supervision	Align clinical teaching with communicative rationality	Documented feedback on communication during clinical observation

Table 3 translates the theoretical framework into concrete educational strategies and assessment outputs for medical training.

## 6 Brazilian Context: DCN 2025 as a Mandate for Communicative Education

Brazil's Resolution CNE/CES n° 3, de 30 de setembro de 2025, explicitly defines the physician as a professional with general, solid, critical, and reflective formation. It requires devotion to principled and humanistic principles [19]. The resolution requires care oriented to the person, family, and community. It asks for respect for diversity and attention to social determinants [19]. It also speaks in the language of competencies. The guidelines require active pedagogies, integrated curricula, and institutional support for student physical health, including protected ‘janelas curriculares’ [19]. These are not minor administrative details; they acknowledge that learning occurs under conditions that can be humanising.

A practical example of these competencies integrated through active pedagogy could be a small‐group session in which students work through a case study of a patient presenting poorly controlled diabetes. In this session, students are guided to link clinical reasoning and diagnostic skills, and to explore social determinants that affect disease management, such as income instability, housing, and food security. They are encouraged to practice ‘escuta qualificada’ by listening to the patient's narrative, considering cultural context, and jointly identifying barriers and solutions. This kind of integrated, participatory session illustrates how the new guidelines move beyond theoretical knowledge to foster skills relevant to real‐world practice [19, 20].

What new obligations do these guidelines create for medical schools and educators? Two major implications follow these requirements. First, humanities and narrative medicine coincide with national competencies. They are not peripheral. The DCNs explicitly mention ‘escuta qualificada’ and the need for sensitivity and responsibility. Listening is part of competence, not simply a matter of personality [19]. To ensure that communicative competence, including effective listening, is truly developed rather than assumed, medical schools can adopt specific assessment methods. For example, Objective Structured Clinical Examinations (OSCEs) with standardised patients can require students to demonstrate active listening, empathy, and lucid communication in real‐world scenarios [37]. Additionally, tools such as the mini‐Clinical Evaluation Exercise (mini‐CEX) allow faculty to directly observe and provide structured feedback to students' communication with patients during real or simulated encounters [38]. These concrete methods make the evaluation of communicative competence measurable and actionable, showing that these requirements can be implemented in practice.

Consider a student sitting with Dona Maria, who has missed several appointments. As she quietly shares, ‘I worry more about my son at home than my own health. Some days I cannot leave him alone,’ the student hears without interrupting, responding only when she finishes. This moment of attentive listening allows confidence and comprehension to emerge, demonstrating how ‘escuta qualificada’ is lived at the bedside.

Second, the DCNs create accountability. If a school claims to be implementing DCN 2025, it must show how it develops and assesses communicative competence. This includes competence in clinical settings and internationally.

This is also an opportunity to connect humanism to the realities of SUS. Person‐centred care in Brazil is inseparable from equity, access, and community context. A Habermasian approach stresses that communicative practice must include disclosure of system constraints. These include exam availability, referral delays, and medication access. For example, when a clinician honestly discusses with the patient that an abdominal ultrasound may require several months of waiting, they can explore interim strategies or options together. This openness fosters trust and helps patients feel respected, even when limitations are necessary. Openness about scarcity is not only practical; it is also a form of respect for persons and a reflection of ethical professionalism. Concealing constraints produces distrust. Transparent negotiation can preserve legitimacy, even when resources are limited [39].

## 7 Discussion

From the perspective developed in this article, clinical reasoning can be understood not only as a technical process of diagnostic inference but also as a communicative practice. Physicians interpret biomedical evidence while simultaneously engaging with the patient's account of illness and navigating institutional expectations embedded in guidelines and healthcare systems. When these elements are integrated through dialogue, clinical reasoning becomes a deliberative activity in which knowledge, norms, and lived experience inform shared decisions about care.

Nostalgic approaches weaken the humanities' critique. Effective critique, as informed by Habermas, looks forward. It diagnoses modernity's distortions and sustains autonomy within complex systems [5]. Health care systems require protocols and documentation to secure safety and equity. Souvatzi et al. argue that addressing moral conflicts in patient care requires a rational approach in clinical bioethics [39]. However, rationality must not override other essential values. Research by Dierkes and colleagues shows that sepsis protocols standardise care and reduce mortality, but clinical outcomes are more closely associated with adequate nurse staffing than with high protocol compliance [40]. According to Hong‐Seon Lee and colleagues, simplified protocols that override clinical judgement can undermine patient autonomy and compromise safety [41].

For educators, these critiques mean that curriculum design should emphasise not only adherence to technical guidelines but also the development of critical thinking and the ability to balance system requirements with patient needs. Concrete teaching strategies could include reflective discussions on the limits of protocols, case studies that ask students to weigh autonomy against standardisation, and training that prioritises both the rationale behind protocols and the clinical judgement needed to apply them wisely.

To address this challenge, two parallel reforms are necessary. The first is educational: clinicians must be trained to sustain deliberation under constraint. Here, communication training must be reframed from politeness to competence, understood as the ability to demonstrate specific micro‐skills in actual interactions. For instance, clinicians should be able to explicitly set a shared agenda with patients under time pressure, summarise and clarify patient perspectives to signal active listening, and negotiate follow‐up and task prioritisation transparently when time is limited. Curriculum examples include simulated patient encounters focused on agenda‐setting, practical workshops to practice real‐time summarisation and negotiation during brief consultations, and reflective exercises using video recordings of actual patient interactions. Educators can use targeted assessment methods, such as structured observation checklists to evaluate agenda‐setting and summary skills, role‐play scenarios with time constraints, and peer feedback on communication effectiveness. Mini‐Clinical Evaluation Exercises (mini‐CEX) and Objective Structured Clinical Examinations (OSCEs) can also be adapted to specifically test a clinician's ability to engage in deliberate, patient‐centred communication under pressure. According to Albury et al., the purposes and concepts of deliberative organisational routines can also support deliberation in challenging situations. Additionally, workflows should be structured to help safeguard attention. For example, if documentation demands exceed human capacity, communicative action becomes impossible [42]. In such cases, the moral burden shifts unfairly to clinicians, who then experience burden and moral injury [43]. Education alone cannot fix this, but it can create professionals able to name the problem and advocate for redesign.

The humanities warn against blindly accepting patient‐centred rhetoric, which can be co‐opted. For example, a patient with a chronic illness at a large urban hospital was informed of the institution's commitment to ‘patient‐centred care.’ In reality, her appointments were rushed to meet throughput targets, and she saw a different clinician at each visit. Jiang et al. note that while pressure injury care has advanced, important gaps persist. Efforts to individualise care often lead to reduced continuity and more demands on patients and clinicians. Institutions may call themselves ‘patient‐centred’ while focusing on throughput, reducing continuity, and turning ‘experience scores’ into commodities. Through a Habermasian lens, this is system colonisation in language: communicative ideals are used to serve bureaucratic aims. The needed response is critical literacy. Students and clinicians must learn to distinguish genuine ‘patient‐centred care’ from branding strategies. In practice, educators can foster critical literacy by asking learners to review actual policy statements or marketing materials, identify examples of authentic versus co‐opted patient‐centred language, and discuss the impact of organisational priorities on patient experience. Classroom activities might include comparing anonymized case studies in which the term ‘patient‐centred care’ is used to mask throughput pressures, or engaging learners in role‐play to practice identifying and addressing discrepancies between stated values and observed practices. In clinical placements, reflective writing prompts can help students analyse moments when patient‐centred ideals are genuinely realised versus when they are superficial. These exercises support students in applying a critical lens and building the discernment needed for authentic, communicative care.

The physician–patient relationship is about social trust, not only individual virtue. Trust is built through communication. It depends on truthfulness, fairness, and perceived sincerity. Yet, some organisational practices can weaken the dialogue that builds trust. For example, strict productivity metrics can force clinicians to rush, thereby limiting opportunities for genuine deliberation. Long shifts and unpredictable schedules reduce continuity, leaving clinicians too tired to engage with patients. A review by Hammad et al. shows that, even with policies in place, committed professionals struggle to maintain communication that sustains trust [44]. Moral shortcomings often stem from systemic limits to open deliberation, not personal failures. Medical education must have a civic function: it trains professionals to sustain legitimacy in healthcare, especially in situations of inequality or limited resources.

## 8 Ethical Limits and Research Horizons

This discussion is conceptual and not based on new empirical research. They help build patient‐centred shared decision‐making, but are not complete solutions for clinical communication. Future research should closely link communication models to both learner and patient results. For example, evaluating how DCN 2025 changes communication instruction in Brazilian schools could assess students' ability to set shared agendas, summarise concerns, and negotiate follow‐up within time limits. It could also be linked to patient satisfaction and a sense of involvement. Assessing narrative medicine curricula could track students' skills in active listening and empathy, as well as patient reports of feeling heard. Studying EHR workflows could examine outcomes such as increased patient turn‐taking, more joint goal‐setting, and greater clinician–patient dialogue. By linking research to clear communication behaviours and outcomes, empirical work can clarify the benefits of these frameworks. Specific research questions that could guide such studies include: ‘How does the incorporation of structured agenda‐setting training into medical curricula affect students' ability to establish shared agendas with patients, as measured by OSCE performance and patient feedback?’ and ‘To what extent does enhanced reflective writing in clinical education improve students' recognition and management of conflicts between system protocols and patient autonomy, as evaluated by qualitative analysis of student reflections and simulated case performance?’ Potential study designs might include pre‐post intervention trials in educational settings, longitudinal studies tracking the development of communication skills, or mixed‐methods research combining quantitative outcome measures with in‐depth qualitative interviews with both learners and patients.

The framework developed here also offers a practical lens for evaluating clinical encounters. From this perspective, the quality of a consultation cannot be assessed solely by diagnostic accuracy or guideline adherence. Instead, clinical practice may be understood as successful when biomedical evidence, institutional constraints, and the patient's narrative are integrated within a communicative process that allows participants to deliberate about the meaning and implications of illness. When one of these domains dominates – whether biomedical reductionism, institutional rule‐following, or purely subjective interpretation‐ the communicative balance of the encounter is disrupted. Viewing consultations through this lens, therefore, provides a conceptual tool for examining how contemporary healthcare systems shape clinical reasoning and the conditions under which meaningful doctor–patient dialogue can occur.

## 9 Conclusion

In conclusion, the doctor–patient relationship is not just a leftover from pre‐technological medicine. It is the ethical and epistemic core of clinical practice. Habermas' theory shows why: clinical action relies on communication where facts, norms, and lived experience are discussed and negotiated. Narrative medicine and the humanities offer ways to strengthen this. Shared decision‐making puts it into practice. Brazil's DCN 2025 policy requires these skills in medical education. The main challenge is alignment. Curricula, assessment, faculty development, and workflows must all support communicative rationality. It should not be taught as an ideal but undermined in clinics. As a first step, institutions could create ‘documentation‐light’ clinics with limited paperwork. This would let teams test workflows focused on dialogue and discussion. Evaluating precision medicine pilot programs helps find barriers, supports, and drives change. By running targeted pilots, especially for biomarker‐guided treatments in places like Brazil, it becomes easier to support patient‐centred, communicative clinical practices.

Although some of these recommendations are grounded in Brazil's policy context, many can be adapted or extended to diverse healthcare systems elsewhere. For example, curriculum reforms that emphasise communicative competence, critical literacy, and the practical testing of workflow changes are relevant in medical education worldwide. Institutions in other countries might tailor ‘documentation‐light’ clinic pilots or critical literacy exercises to fit their own regulatory and organisational realities. By focusing on the underlying principles of communicative rationality and aligning education with clinical practice, educators internationally can foster meaningful doctor–patient dialogue and patient‐centred care within their distinct contexts.

## Funding

The author has nothing to report.

## Ethics Statement

1

This study is a narrative review of previously published literature. No human participants, patient data, or identifiable personal information were involved. Therefore, according to institutional and international research ethics guidelines, formal ethical approval was not required.

## Conflicts of Interest

The author declares no conflicts of interest.

## Data Availability

The data that support the findings of this study are available from the corresponding author upon reasonable request. No datasets were generated or analysed during the current study. All information discussed in this manuscript is derived from published literature cited in the reference list.
